# ﻿*Primulalizipingensis* (Primulaceae), a new species from Sichuan, China

**DOI:** 10.3897/phytokeys.236.112169

**Published:** 2023-12-14

**Authors:** Wen-Bin Ju, Liu-Yang He, Qi Lan, Ying-Hao Wu, Heng-Ning Deng, Xing-Jin He, Xin-Fen Gao, Bo Xu

**Affiliations:** 1 China-Croatia “Belt and Road” Joint Laboratory on Biodiversity and Ecosystem Services, Key Laboratory of Mountain Ecological Restoration and Bioresource Utilization & Ecological Restoration Biodiversity Conservation, Chengdu Institute of Biology, Chinese Academy of Sciences, Chengdu 610041, Sichuan, China Chengdu Institute of Biology, Chinese Academy of Sciences Chengdu China; 2 Key Laboratory of Bio-Resources and Eco-Environment of Ministry of Education, College of Life Sciences, Sichuan University, Chengdu 610065, Sichuan, China Sichuan University Chengdu China; 3 University of Chinese Academy of Sciences, Beijing 100049, China University of Chinese Academy of Sciences Beijing China; 4 Management Bureau of Liziping National Nature Reserve, Shimian 625400, Sichuan, China Management Bureau of Liziping National Nature Reserve Shimian China

**Keywords:** *Aleuritia*, Hengduan Mountains, *Primula* sect, taxonomy

## Abstract

A new species, *Primulalizipingensis* W.B.Ju, L.Y.He & X.F.Gao, found in Shimian County, Sichuan, China, is described and illustrated. It is morphologically similar to *P.rhodochroa* and *P.socialis*, but can be distinguished from them in having shorter plants covering with white farinose, leaf margin sharply dentate above the middle, the leaf blade becomes papery after drying, scapes obsolete, the bract linear-lanceolate to subulate, solitary at the base of the pedicel, and the white hairs present inside the corolla tube.

## ﻿Introduction

The genus *Primula* L. is one of the largest genera in the Primulaceae with more than 500 species, and widely distributed in the temperate and alpine regions of the Northern Hemisphere with its major concentration in the Sino-Himalayan regions and in western China, with only a few occurring on mountains in Ethiopia, tropical Asia and South America (Hu 1990, 1994; Hu and Kelso 1996; APG 2016). In China, more than 300 species of *Primula* have been recorded, which are concentrated in the southwestern and northwestern provinces, with only a few species distributed in other regions (Hu and Kelso 1996; Richards 2003). Sichuan Province is a particularly important biodiversity hotspot in China. The southwestern area of Sichuan Province belongs to the Hengduan Mountains, which is recognized as one of the 36 biodiversity hotspots in the world. Moreover, this region serves as a center of diversity for *Primula* (Hu 1994; Hu and Kelso 1996). Since the publication of the Flora of China, numerous new species of *Primula* have been discovered and described in the region (Hu and Geng 2003; Wu et al. 2013; Xu et al. 2014, 2015a, 2015b, 2016, 2017, 2019; Ju et al. 2018, 2021; Yuan et al. 2018; Li et al. 2023).

In May 2023, during field expeditions in Liziping National Nature Reserve, an unusual *Primula* population was discovered. Upon observing its morphological characteristics, such as its mealy, deciduous perennials, and overwintering through an above-ground mealy resting bud, along with bracts that are typically smaller and somewhat swollen or thick, calyx prominently 5-veined, corolla narrow tube and lobes apex deeply emarginate, we have identified this species as belonging to the PrimulasectionAleuritia. After consulting relevant literature (Smith and Word 1926; Smith and Fletcher 1944; Hu 1986, 1990; Fang 1994, 2003; Hu and Kelso 1996; Wu 1999; Richards 2003) and herbarium specimens (BM, E, K, KUN, and US), we have concluded that this species is indeed unique and previously undescribed, and similar to *Primularhodochroa* W.W.Sm. and *P.socialis* F.H.Chen & C.M.Hu. Therefore, it is described here as a new species.

## ﻿Materials and methods

The descriptions and illustrations presented here were based on an analysis of the habits and characteristics observed in fresh material during field surveys, as well as the examination of type specimens deposited in CDBI. The morphological features of this new species, as well as those of its similar species, were described using the terminology outlined in the Flora of China (Hu and Kelso 1996). To supplement our examination, we accessed digital specimens online through various platforms, including the Chinese Virtual Herbarium (http://www.cvh.ac.cn/), JSTOR Global Plants (https://plants.jstor.org/), the Global Biodiversity Information Facility (https://www.gbif.org/), and Europeana (https://www.europeana.eu), with particular emphasis on type specimens from BM, E, K, KUN, and US. The regional conservation status was assessed following the IUCN guidelines (IUCN Standards and Petitions Committee 2022).

## ﻿Taxonomic treatment

### ﻿ Primula lizipingensis

Taxon classificationPlantaeEricalesPrimulaceae

W.B.Ju, L.Y.He & X.F.Gao sp. nov.

7BC63FBB-BB59-581A-905E-E29C45AFA090

urn:lsid:ipni.org:names:77332792-1

Figs 1 , 2 , 3

#### Diagnosis.

*Primulalizipingensis* is morphologically similar to *P.rhodochroa* and *P.socialis*. However, the new species can be easily distinguished from *P.rhodochroa* by its leaf margin sharply dentate above the middle, scape absent, flower solitary subtended by a single, linear-lanceolate to subulate bract, the calyx lobes split to the middle, the corolla tube longer than the calyx, and its interior has white hairs. Compared with *P.socialis*, the new species is covered with white farinose (vs. glabrous), leaf oblanceolate to spathulate, and papery when dry (vs. obovate-elliptic to oblanceolate, membranous when dry), bracts linear-lanceolate to subulate (vs. linear), calyx lobes split to the middle (vs. split to the middle or below), and corolla tube hairy inside (vs. glabrous).

**Figure 1.**
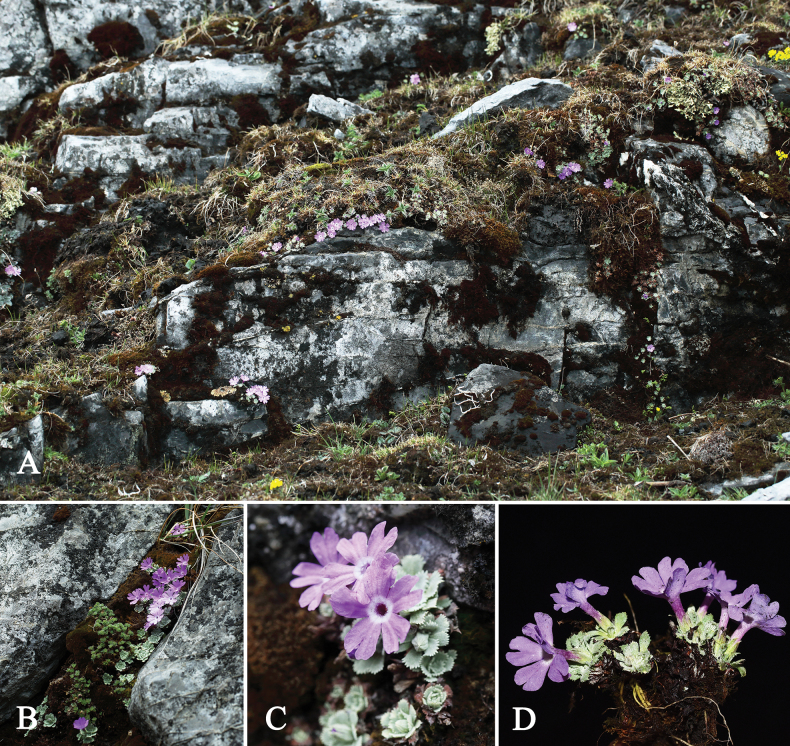
Habitat of the *Primulalizipingensis* sp. nov. (**A–D**).

#### Type.

China. Sichuan: Shimian county, Liziping National Nature Reserve, growing in moist rock crevices covered with moss; 29°00′N, 102°11′E, 4318 m alt., 18 May 2023 (fl.), *Liuyang He J-1201* (holotype CDBI!; isotypes KUN!)

**Figure 2.**
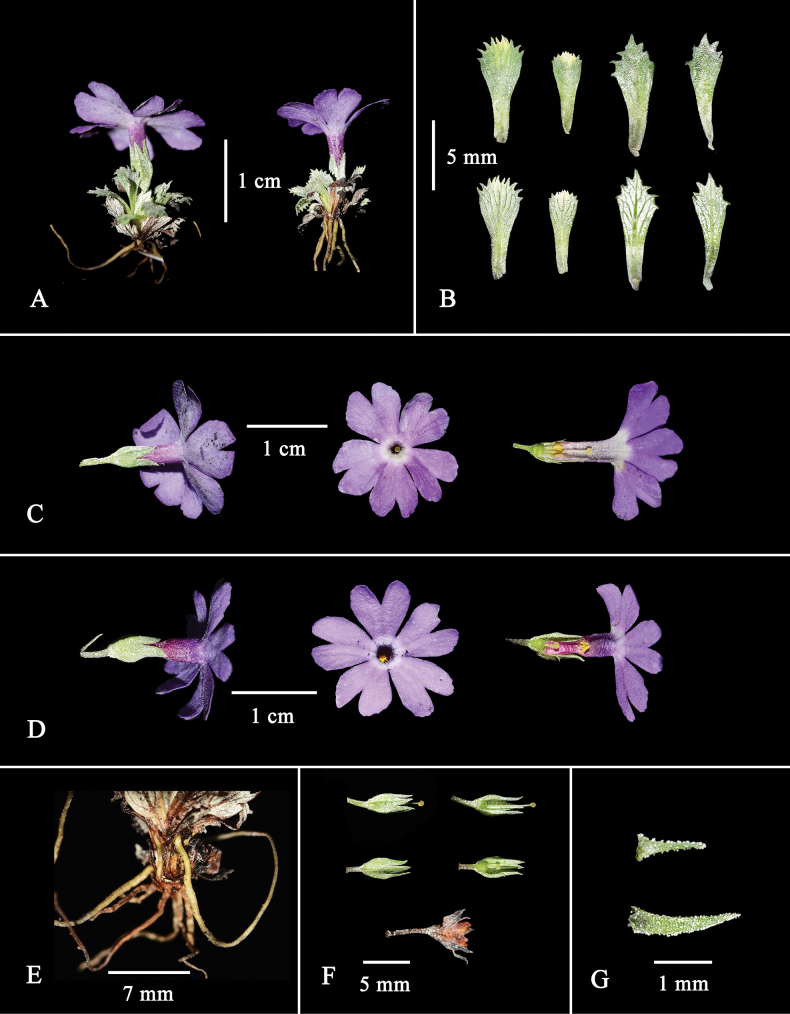
*Primulalizipingensis* sp. nov. **A** fresh plants **B** leaves **C** pin flower (showing flower side and front, the position of anthers and styles in the tube) **D** thrum flower (showing flower side and front, the position of anthers and styles in the tube) **E** plants base **F** calyx, capsule and mature fruit that has already split **G** bracts. Photographs by W-BJ.

#### Description.

A dwarf farinose tufted perennial herb, at most 2.5 cm tall, with a short stout rhizome and covered at the base by the withered remains of old leaves of the previous year. ***Leaves*** forming a dense tuft, papery when dry, including the petiole 5–12 mm long, 2.5–5.5 mm broad, oblanceolate to spathulate, rounded or acute at apex, tapering into the papery winged petiole which when fully developed is as long as the leaf blade, margin sharply dentate above the middle, green above with a thin covering of potentially white farinose glands, thickly covered below with white farina, the midveins and lateral veins are prominent at abaxially. ***Scape*** almost obsolete, bearing one flower. ***Bracts*** solitary, at base, linear-lanceolate to subulate, more or less white farinose, 1–2.8 mm long. ***Flower*** solitary, heterostylous. ***Pedicels*** 1.5–5.0 mm long, cover the white farinose, not extended in fruit. ***Calyx*** green, campanulate, 5–5.5 mm long, farinose both within and without, prominently 5-veined, split to the middle, lobes narrowly triangular to lanceolate, apex acute. ***Corolla*** obscurely annulate, with sparsely white farinose glands abaxially; limb 14–18 mm across, funnelform; tube deep purple, 1.3‒1.8 times the length of the calyx and a few white hairs adaxially; lobes pale purple with a white eye, spreading, 6.5–8.2 × 4.5–6.0 mm, broadly obovate, deeply emarginate. ***Pin flowers***: corolla tube 7–8 mm long, widely ampliated above the insertion of stamens; stamens ca. 1.5 mm above base of corolla tube; style ca. 2/3 as long as tube. ***Thrum flowers***: corolla tube ca. 8 mm long, widely ampliated above insertion of stamens; stamens inserted slightly above the middle of corolla tube; style ca. 2 mm. ***Capsule*** oblong, slightly shortly than the calyx.

**Figure 3.**
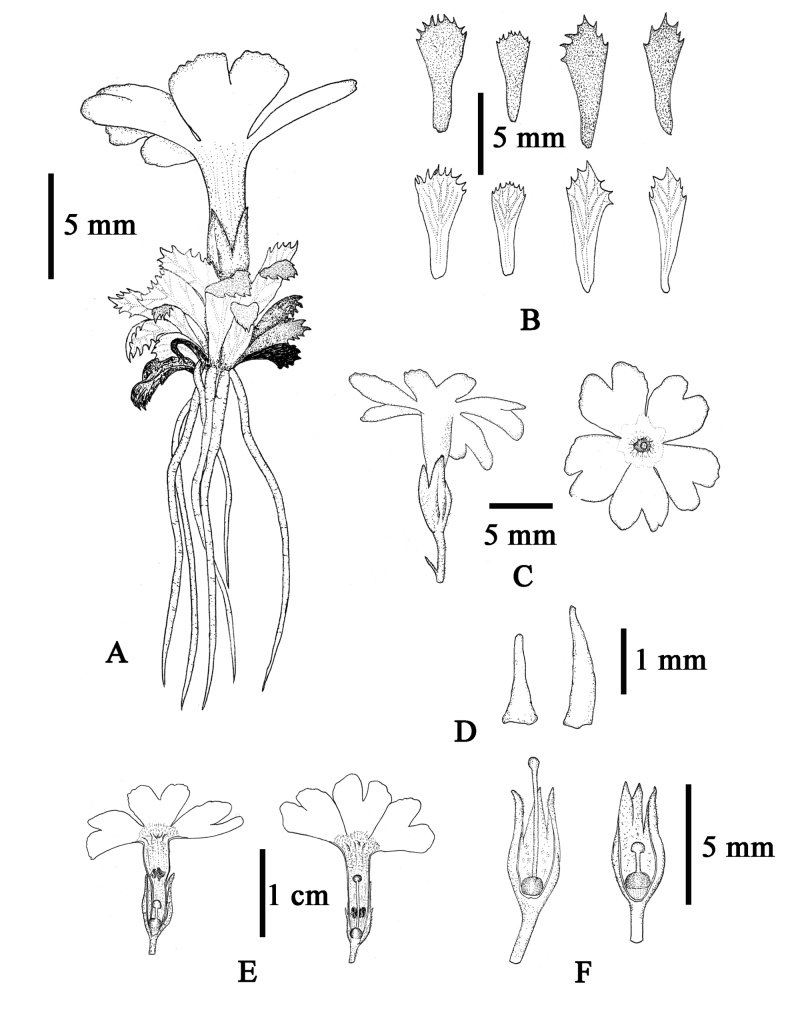
*Primulalizipingensis* sp. nov. **A** habit **B** leaves **C** inflorescence and front of the flower **D** bracts **E** pin flowers (left) and thrum flowers (right) **F** calyx and pistil of pin flowers (left) and thrum flowers (right). Drawn by Z-LL.

#### Phenology.

Flowering occurs in May, fruiting is unknown.

#### Distribution and habitat.

*Primulalizipingensis* is only known from the type locality Liziping National Nature Reserve, Shimian County, Sichuan, China. It grows in moss-covered limestone crevices at an altitude of 4300–4400 meters.

#### Etymology.

The specific epithet ‘lizipingensis’ refers to the type locality where the new species occurs, Liziping National Nature Reserve, Sichuan, China.

#### Conservation status.

Data Deficient (DD). Due to insufficient field investigations, we do not yet have a clear understanding of its natural distribution and population status, nor do we have enough information to directly or indirectly assess its risk of extinction. Therefore, we temporarily categorize this species as Data Deficient according to the International Union for Conservation of Nature Red List Categories (IUCN Standards and Petitions Committee 2022). Further field investigations in the high-altitude areas of western Sichuan in the future can provide more information about its abundance and distribution.

#### Discussion.

The section Aleuritia was originally considered by Duby (1844) with citation of type species *P.farinosa* L. This section is a large group having more than 80 species with wide distribution. The distribution is almost throughout the range of the genus *Primula*, spanning across circum-arctic regions and major mountain systems in Europe, North America, and Asia (Hu 1990, 1994; Hu and Kelso 1996). Notably, this is the sole section of the *Primula* genus that includes representative species in South America (Hu 1994; Basak et al. 2014). In this study, we followed Hu’s (1990) taxonomic treatment of the *Primula* in China.

Further research indicates that this new species is similar to *P.rhodochroa* and *P.socialis* in that they have dwarf farinose plant, possess short rhizomes, and produce solitary flowers emerging from basal rosettes. *P.rhodochroa*, distributed in southeast Xizang, thrives in wet moss on boulders or rock faces at altitudes of 4000‒5000 meters. *P.socialis*, found in western Yunnan, flourishes in shady crevices of mountain rocks at an altitude of 2950 m. *P.lizipingensis*, found in Shimian County, Sichuan Province, on the eastern edge of the Hengduan Mountains, grows in moss-covered limestone crevices at an altitude of 4300–4400 m. Despite all three growing in limestone dam crevices with moss, prolonged geographical isolation has led to morphological differentiation, resulting in the emergence of distinct species. The same situation also occurs in the morphological and habitat similarities between *P.kialensis* Franchet and P.yunnanensissubsp.membranifolia (Franchet) Halda. These species are both covered with yellow farinose and showing resemblances in leaf morphology, and inflorescence. The main difference is that P.yunnanensissubsp.membranifolia has a corolla tube length 2–4 times that of the calyx, and its leaves texture is membranous, whereas *P.kialensis* has a corolla tube length 1–2 times that of the calyx and chartaceous leaves. The former is distributed in counties such as Jiulong, Kangding, and Lixian, located in the eastern part of the Hengduan Mountains, and belongs to a unique species in Sichuan. The latter is distributed in counties such as Dali, Yangbi, and Fengqing, located in the central and western parts of the Hengduan Mountains, and belongs to a unique species in Yunnan. The new species differs from *P.rhodochroa* in the characteristic of leaf margin teeth, the presence or absence of scape, the characteristic and numbers of bracts, the split degree of calyx lobes, and the presence of hairs inside the corolla tube. Morphologically, the new species is more closely related to *P.socialis* because both are characterized by having solitary flower and bract, without a scape, but easily recognized by the present of white farina covering the whole plant, leaves smaller and papery after drying with margin sharply dentate, the bract linear-lanceolate to subulate, and hairy with white hairs inside the corolla tube. A detailed comparison of the three species is shown in Table 1.

**Table 1.** Comparison of morphological characters among *Primulalizipingensis*, *P.rhodochroa* and *P.socialis*.

Characters	Species		
	*Primulalizipingensis*	*P.rhodochroa*	*P.socialis*
Farinose color	White	white	yellow
Leaf shape	oblanceolate to spathulate	oblanceolate to narrowly obovate	obovate-elliptic to oblanceolate
Leaf blade	margin sharply dentate above the middle	margin denticulate to dentate	margin dentate above the middle
	papery when dry	papery when dry	membranous when dry
	rounded or acute at apex	obtuse to rounded at apex	obtuse to rounded at apex
Scape	Absent	present but less than 1 cm	absent
Inflorescence	flowers solitary	1−2(4)-flowered umbel	flowers solitary
Bracts	1, linear-lanceolate to subulate	2−3, linear	1, linear
Calyx lobes	split to the middle of the calyx	split to 1/3 of the calyx	split to the middle or below of the calyx
Corolla	tube longer than the calyx, and white hairs adaxially	tube ca. as long as calyx, glabrous adaxially	tube longer than the calyx, glabrous adaxially

## Supplementary Material

XML Treatment for Primula lizipingensis

## ﻿Additional information

### Author ORCIDs

Wen-Bin Ju https://orcid.org/0000-0002-5665-6290

Heng-Ning Deng https://orcid.org/0000-0002-0581-0649

Xing-Jin He https://orcid.org/0000-0003-2064-0112

Xin-Fen Gao https://orcid.org/0000-0002-5703-1639

Bo Xu https://orcid.org/0000-0003-3507-9321

### Data availability

All of the data that support the findings of this study are available in the main text.
